# TLR4 deficiency reduces pulmonary resistance to *Streptococcus pneumoniae* in gut microbiota-disrupted mice

**DOI:** 10.1371/journal.pone.0209183

**Published:** 2018-12-18

**Authors:** Hongyan Wang, Pengjing Lian, Xiaofei Niu, Lihong Zhao, Xiang Mu, Bo Feng, Jingyun Li, Zhenni Liang, Jian Qiao

**Affiliations:** 1 Department of Pathophysiology, College of Veterinary Medicine, China Agricultural University, Beijing, China; 2 Department of Veterinary Medicine, College of Life Sciences and Food Engineering, Hebei University of Engineering, Handan, China; 3 Beijing Key Laboratory of Traditional Chinese Veterinary Medicine, Animal Science and Technology College, Beijing University of Agriculture, Beijing, China; University of Illinois at Urbana-Champaign, UNITED STATES

## Abstract

*Streptococcus pneumoniae* is a clinically important pathogen responsible for significant morbidity and mortality worldwide. Disruption of the host gut microbiota by antibiotics reduces the pulmonary resistance to *S*. *pneumoniae*. The aim of our study was to determine the potential role of TLR4 in the reduced pulmonary resistance to *S*. *pneumoniae* following gut microbiota disruption. Wild-type and TLR4-deficient mice were given broad-spectrum antibiotics for 3 weeks by oral gavage to disrupt the gut microbiota, and subsequently inoculated intra-nasally with *S*. *pneumoniae*. The extent of the decline in pulmonary resistance in both animal groups was evaluated in terms of the overall survival and pulmonary bacterial clearance. Both survival and pulmonary clearance of *S*. *pneumoniae* were lower in the TLR4-deficient mice with disrupted gut microbiota, compared to their intestinally healthy counterparts after pneumococcal infection. However, the degree of decline was much lower in the TLR4-deficient mice compared to the wild-type mice. Our findings indicate that impaired TLR4 function might be the basis of the reduced pulmonary resistance to *S*. *pneumoniae* caused by gut microbiota disruption.

## Introduction

*Streptococcus pneumoniae* is the causative agent of a wide range of diseases, such as pneumonia, otitis media, bacteremia, pleurisy, and meningitis [1–3]. Every year, around 2 million people worldwide die from pneumococcal diseases [1], with pneumonia presenting the major public health challenge [4, 5]. Vaccines and antibiotics have been used for many years to manage and control *S*. *pneumoniae* infections [6–8]. However, the evolution of *S*. *pneumoniae* under selective pressure of vaccines [1], and the emergence of multidrug-resistant pneumococcal strains by excessive and inappropriate use of antibiotics have made both strategies challenging [8–10]. To develop new treatment strategies therefore, a better understanding of the pathogenic mechanisms of *S*. *pneumoniae* is needed.

Previous studies have shown that disruption of the gut microbiota due to the long-term use of broad-spectrum antibiotics not only weakens the intestinal immune barrier, but also reduces the pulmonary resistance against pathogens like the influenza virus, *Staphylococcus aureus*, *Escherichia coli*, *Klebsiella pneumoniae*, *Burkholderia pseudomallei* and *S*. *pneumoniae* [11–19]. Under physiological conditions, the gut microbiota-derived ligands are constantly translocated across the intestinal mucosa into the bloodstream, and identified by the systemic pattern recognition receptors (PRRs) expressed on the neutrophils, macrophages and dendritic cells in target tissues, resulting in their activation [20]. Disruption of the gut microbiota by broad-spectrum antibiotics reduces the concentration of gut microbiota-derived PRR-ligands, which eventually impairs the antimicrobial capacity of immune cells bearing PRRs [13, 15, 19, 20].

Toll-like receptor 4 (TLR4) is an important PRR involved in the innate immune response to pneumolysin produced by *S*. *pneumoniae* [21–27]. In recent years, TLR4 has been found to be associated with the lung immunity regulated by gut microbiota. Studies have implicated TLR4 in the decreased pulmonary resistance to *E*. *coli* or *K*. *pneumoniae* in gut microbiota-disrupted or germfree mice [14, 28], since the impaired immune response in these mice can be reversed with the TLR4 ligand lipopolysaccharide (LPS) [14, 28]. Schuijt TJ *et al* found that the mortality rate and bacterial load were higher in gut microbiota-disrupted mice compared to the undisrupted controls after pneumococcal infection, and the alveolar macrophages and whole-blood neutrophils isolated from the former were hypo-responsive to LPS compared to that from control mice [17]. We hypothesized therefore that the levels of the TLR4 ligands are decreased in mice with disrupted gut microbiota, which is the basis of the reduced pulmonary resistance to *S*. *pneumoniae* seen in these mice.

To prove this hypothesis, we evaluated whether TLR4 deficiency affected the pulmonary resistance of gut microbiota-disrupted mice to *S*. *pneumoniae* infection. We found that the survival and pulmonary bacterial clearance were lower in the gut microbiota-disrupted TLR4-deficient mice compared to the undisrupted controls after pneumococcal infection, but the degree of decline was much lower compared to the wild-type mice. This clearly indicates that decreased pulmonary resistance due to gut microbiota disruption is associated with impaired TLR4 function. Our study provides an experimental basis for novel therapeutic strategies against pneumococcal infection that exploit the TLR4 pathway.

## Materials and methods

### Mouse strains

Specific pathogen-free, 6–8 week-old male wild-type (C3H/HeN) mice were purchased from the Beijing Vital River Laboratory Animal Technology Company Limited (China), and the corresponding TLR4-deficient (C3H/HeJ) mice were obtained from the Model Animal Research Center of Nanjing University (China). The TLR4-deficient mice have a spontaneous mutation in the third exon of the *Tlr4* gene (Pro712His), which yields a nonfunctional TLR4 [29]. All mice were acclimatized for 2 weeks prior to the experiments. All research personnel received training in animal care or handling, and all experiments were approved by the Laboratory Animal Welfare and Animal Experimental Ethical Committee of the China Agricultural University (No. CAU20180628-2 and No. CAU20181008-1).

### Bacterial strains

Wild-type *S*. *pneumoniae* strain D39 (NCTC 7466, serotype 2) [17, 21, 26] was obtained from the Chongqing Medical University (China), and cultured in THY broth (Todd-Hewitt broth supplemented with 0.5% yeast extract) at 37°C under 5% CO_2_ [26]. To eliminate the effect of ampicillin, one of drugs used for gut microbiota disruption in our study, on *S*. *pneumoniae*, an ampicillin-resistant strain was obtained to induce pneumonia in the mice. The minimal inhibitory concentration (MIC) of ampicillin against *S*. *pneumoniae* was first determined by the broth microdilution method, and drug resistance was induced *in vitro* as described previously [30]. Briefly, *S*. *pneumoniae* culture was serially passaged in the presence of ampicillin till a 32-fold increase in the MIC was achieved, and the ampicillin-resistant *S*. *pneumoniae* strain was obtained.

The inoculum size of *S*. *pneumoniae* for intranasal infection was determined by established protocols. Briefly, *S*. *pneumoniae* was cultured until mid-log phase (OD_600_ = 0.4–0.5), harvested by centrifuging at 3000 × *g* for 10 minutes at 4°C, and washed twice with sterile phosphate-buffered saline (PBS) [31]. The bacteria were re-suspended in PBS at different concentrations, and inoculated intra-nasally into wild-type mice lightly anesthetized with isoflurane to determine its median lethal dose [32]. We used 0.2 median lethal doses (2.3 × 10^8^ colony-forming units) of *S*. *pneumoniae* in our study.

### Gut microbiota disruption with broad-spectrum antibiotics

Gut microbiota is experimentally disrupted by administering broad-spectrum antibiotics (ampicillin, neomycin, metronidazole and vancomycin) in the drinking water [12, 13, 15, 17]. Since this route of administration would result in severe dehydration and subsequently affect the host immunity [33, 34], we used a modified protocol. The mice were given 0.2 ml of the following broad-spectrum antibiotics– 10 mg/ml ampicillin (Amresco), 10 mg/ml neomycin sulfate (Amresco) and 5 mg/ml metronidazole (Hualu Holding Co., Ltd.)–twice daily for 3 weeks by oral gavage. Vancomycin was excluded since it might have had an impact on the pneumococcal infection. To ensure that the other antibiotics had no effect on pneumococcal infection, and to test any potential antibacterial effect of murine blood and tissues, *S*. *pneumoniae* was co-cultured with the sera, and the lung or liver homogenates of the gut microbiota-disrupted mice, prior to initiating the infection (S1 Table).

### *S*. *pneumoniae* inoculation and experimental design

The wild-type and TLR4-deficient mice were each randomized into the following four groups: Group I—gut microbiota-undisrupted uninfected that received only sterile saline, Group II—gut microbiota-disrupted uninfected that received sterile saline supplemented with antibiotics, Group III—gut microbiota-undisrupted infected that received sterile saline and *S*. *pneumoniae* inoculation, and Group IV—gut microbiota-disrupted infected that received sterile saline supplemented with antibiotics, and *S*. *pneumoniae* inoculation. Three days after cessation of antibiotic administration (see above), the mice were inoculated intra-nasally with sterile PBS or 0.2 median lethal dose of *S*. *pneumoniae*. The survival of the infected mice (n = 20 per group) was recorded for 7 days post-infection, and the mice were weighed and monitored every 3 hours (and every hour when their condition deteriorated) for signs of illness and death. The animals that lost 25% of their original body weight were euthanized. Another cohort (n = 6 per group) was used to analyze the pulmonary bacterial load, cytokine production and lung histopathology as described in the following sections.

### Diversity analysis of gut microbiota after antibiotic treatment

The alpha diversity of the gut microbiota was analyzed on the basis of bacterial 16S ribosomal RNA (16S rRNA) gene sequencing. Fresh feces were collected 3 weeks after antibiotic administration, and 3 days after treatment cessation, and immediately frozen and stored at −80°C. Microbial DNA was extracted from the thawed fecal samples, and the V3–V4 region of the bacterial 16S rRNA gene was amplified by polymerase chain reaction (PCR) using primers 338F (5’-ACTCCTACGGGAGGCAGCAG-3’) and 806R (5’-GGACTACHVGGGTWTCTAAT-3’) [35]. The amplicons were paired-end sequenced on an Illumina MiSeq platform. The operational taxonomic units (OTUs) were clustered with 97% similarity cutoff using UPARSE. The taxonomy of each 16S rRNA gene sequence was analyzed by RDP Classifier against the SILVA 16S rRNA database using a confidence threshold of 70% [36].

### Measurement of pulmonary bacterial load

To determine the effect of gut microbiota on early innate immune response to pneumococcal infection, mice were euthanized 6 hours and 12 hours post-infection by isoflurane overdose [15, 19]. Whole lungs were removed and weighed aseptically, and then homogenized in 1 ml sterile PBS. The volume of the lung homogenate was increased to 3 ml with sterile PBS, and serial 10-fold dilutions were prepared. One hundred microliters of each dilution was spread on blood agar plates, and incubated at 37°C under 5% CO_2_ for 24 hours. The number of colonies were counted and the colony-forming units were calculated to determine the initial bacterial load [31].

### Measurement of cytokine levels in the lungs

Lung homogenates were centrifuged at 4°C and 900 × *g* for 10 minutes, and the supernatants were collected and stored at −80°C until use. The levels of tumor necrosis factor alpha (TNF-α) and interleukin-1 beta (IL-1β), which are important mediators of innate host defense during pneumococcal pneumonia [37–41], were measured using Platinum ELISA Kits (eBioscience, Vienna, Austria).

### Histopathological examination of lung tissue

Mice were euthanized by isoflurane overdose 6 hours and 12 hours after infection. The lungs were removed, fixed with 4% paraformaldehyde and embedded in paraffin. The 5-μm sections were stained with hematoxylin and eosin (H & E) according to standard protocols [31].

### Statistical analysis

All results are presented as means ± standard deviation (SD). Data were analyzed using mothur program v.1.30.1 [42] or IBM SPSS Statistics v20, and the graphics were prepared with STAMP software or GraphPad Prism software v5.0. Statistical significance was determined by the unpaired Student’s t test and log-rank test. P values < 0.05 were considered statistically significant.

## Results

### Antibiotic administration disrupts the gut microbiota

Broad-spectrum antibiotics were administrated to both wild-type and TLR4-deficient mice by oral gavage, and the microbial diversity was analyzed by sequencing the bacterial 16S rRNA genes using fresh feces. The alpha diversity analysis showed that the richness and diversity of the intestinal microbiota were greatly decreased in the gut microbiota-disrupted mice compared to the undisrupted controls (S2 and S3 Tables: P < 0.01). Specifically, the abundance of the Bacteroidetes (S4 and S5 Tables, S1 and S2 Figs: P < 0.05) and Firmicutes phyla (S4 and S5 Tables, S1 and S2 Figs: P < 0.05) were significantly lower, while that of the Proteobacteria phylum was significantly greater in the feces of the gut microbiota-disrupted mice (S4 and S5 Tables, S1 and S2 Figs: P < 0.01). Taken together, long-term antibiotic administration disrupted the gut microbiota in both wild-type and TLR4-deficient mice, in terms of both abundance and species diversity.

### Gut microbiota disruption decreases the pulmonary resistance to *S*. *pneumoniae*

**Fig 1:**
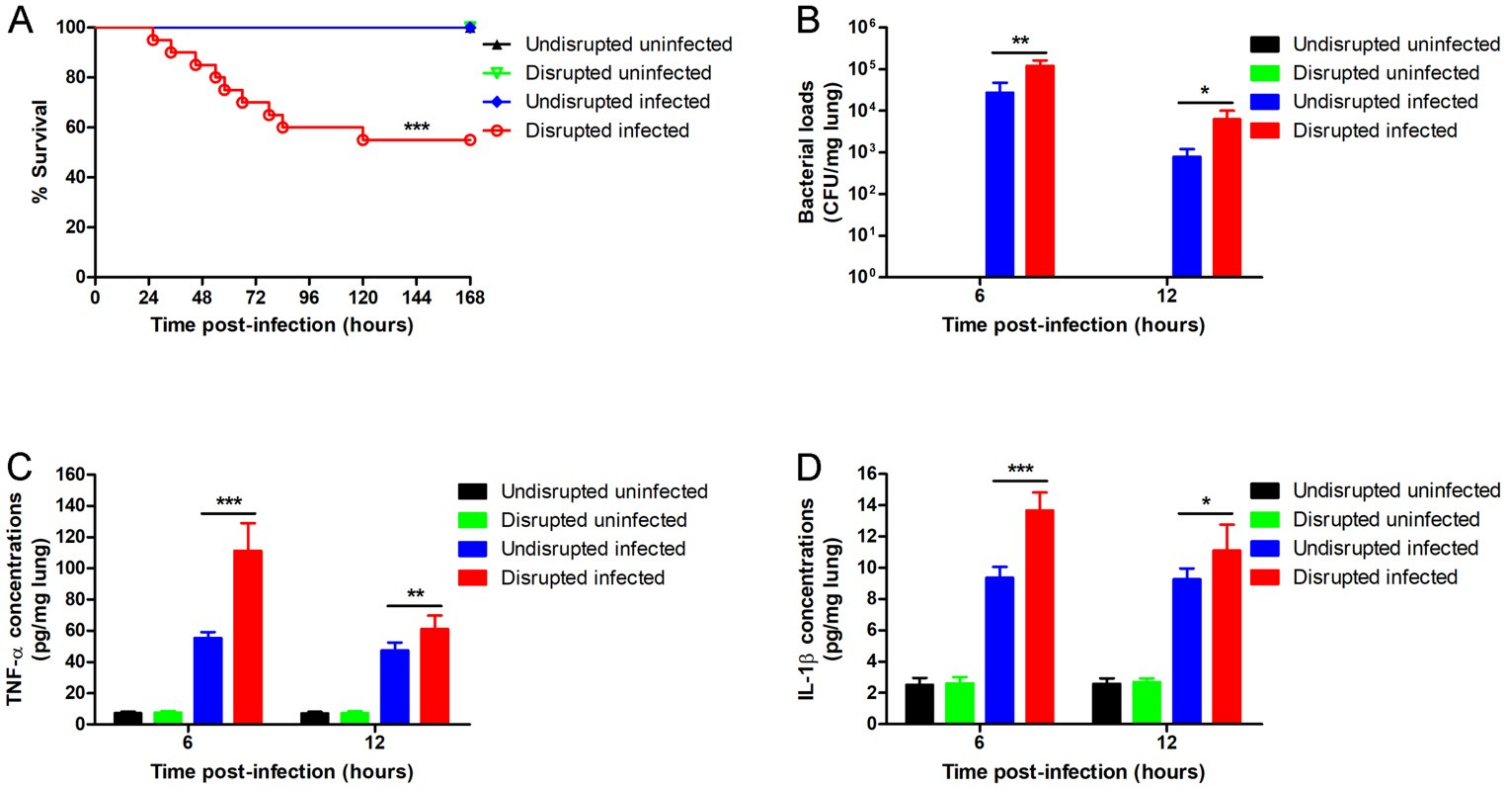
Gut microbiota disruption decreases the pulmonary resistance to *S*. *pneumoniae*. Wild-type mice were given broad-spectrum antibiotics or autoclaved saline. Three days after cessation of administration, mice were inoculated intra-nasally with 2.3 × 10^8^ CFU of *S*. *pneumoniae*. (A) The survival was observed for 7 days (n = 20 per group). (B) Bacterial load, and (C) TNF-α and (D) IL-1β concentration per mg lung tissue were determined 6 hours and 12 hours after infection (n = 6 per group). CFU, colony-forming units. Data are presented as means ± SD. *P < 0.05, **P < 0.01, ***P < 0.001, analyzed with the log-rank test or unpaired Student’s t test.

**Fig 2:**
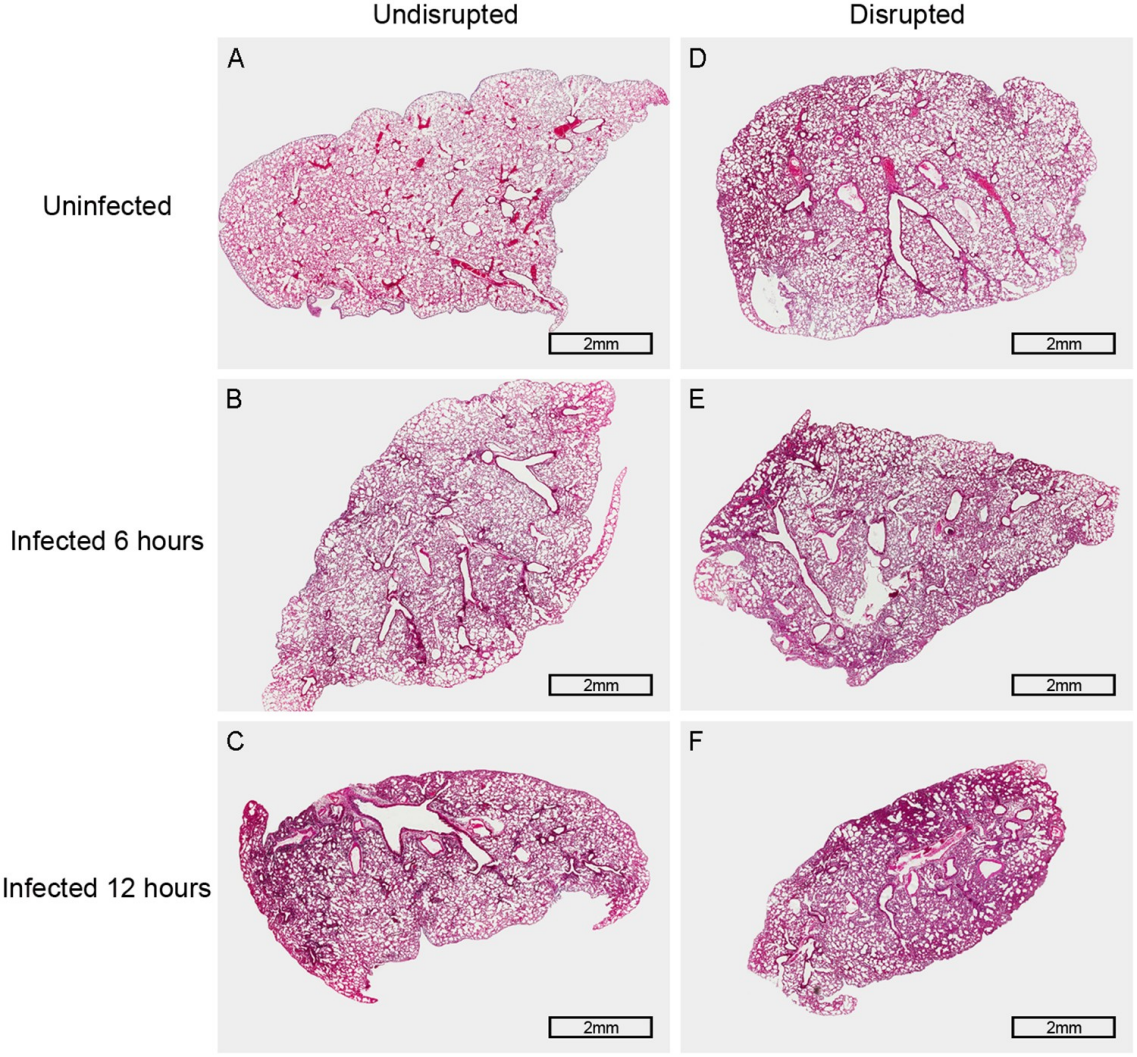
Histopathology of the lung in wild-type mice 6 hours and 12 hours after pneumococcal infection. Representative pictures of H & E stained lung tissues: (A) The picture of the lung tissue in gut microbiota-undisrupted uninfected mice (n = 6 per group). (B and C) Pictures of the lung tissue in gut microbiota-undisrupted mice 6 hours and 12 hours after infection (n = 6 per group). (D) The picture of the lung tissue in gut microbiota-disrupted uninfected mice (n = 6 per group). (E and F) Pictures of the lung tissue in gut microbiota-disrupted mice 6 hours and 12 hours after infection (n = 6 per group). Original magnification, × 20; scale bar = 2 mm.

To test whether gut microbiota disruption affected pulmonary resistance against pneumococcal infection, the wild-type mice were treated with broad-spectrum antibiotics and infected intra-nasally with *S*. *pneumoniae*. The survival (Fig 1A: P < 0.001) and pulmonary bacterial clearance (Fig 1B: P = 0.001, 6 hours; P = 0.016, 12 hours) of the gut microbiota-disrupted mice was reduced significantly compared to the undisrupted controls. In addition, the gut microbiota-disrupted mice showed more severe lung injury compared to the undisrupted controls (Fig 2). Therefore, antibiotic-induced gut microbiota disruption significantly decreased pulmonary resistance to *S*. *pneumoniae*. In addition, pulmonary TNF-α and IL-1β levels of the gut microbiota-disrupted mice were significantly increased (Fig 1C: P < 0.001, 6 hours; P = 0.006, 12 hours. Fig 1D: P < 0.001, 6 hours; P = 0.031, 12 hours) after infection compared to the controls.

### TLR4 deficiency reduces host resistance to *S*. *pneumoniae*

**Fig 3:**
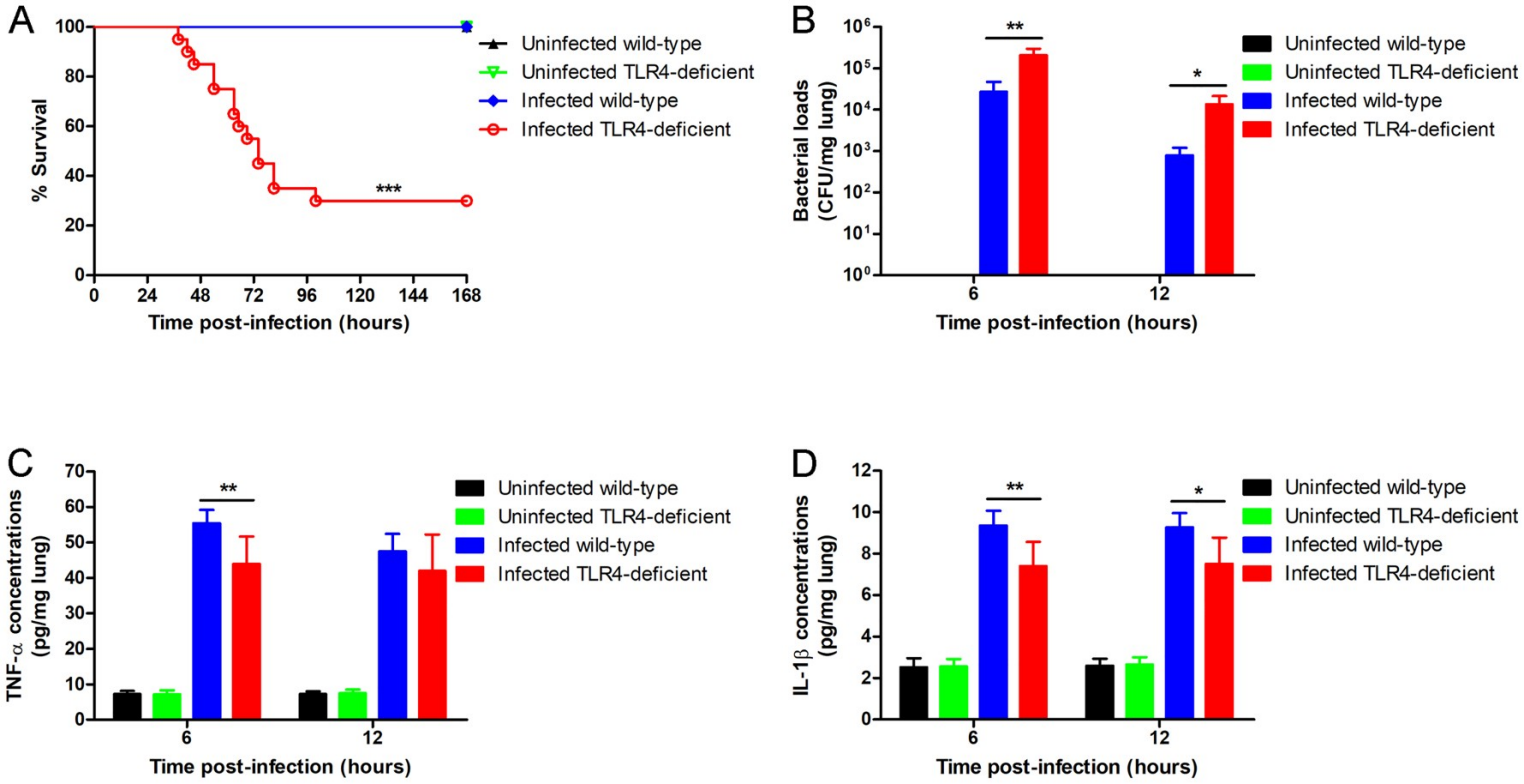
TLR4 deficiency reduces host resistance to *S*. *pneumoniae*. Wild-type and TLR4-deficient mice were intra-nasally inoculated with 2.3 × 10^8^ CFU of *S*. *pneumoniae*. (A) The survival was observed for 7 days (n = 20 per group). (B) Bacterial load, and (C) TNF-α and (D) IL-1β concentration per mg lung tissue were determined 6 hours and 12 hours after infection (n = 6 per group). CFU, colony-forming units. Data are presented as means ± SD. *P < 0.05, **P < 0.01, ***P < 0.001, analyzed with the log-rank test or unpaired Student’s t test.

**Fig 4:**
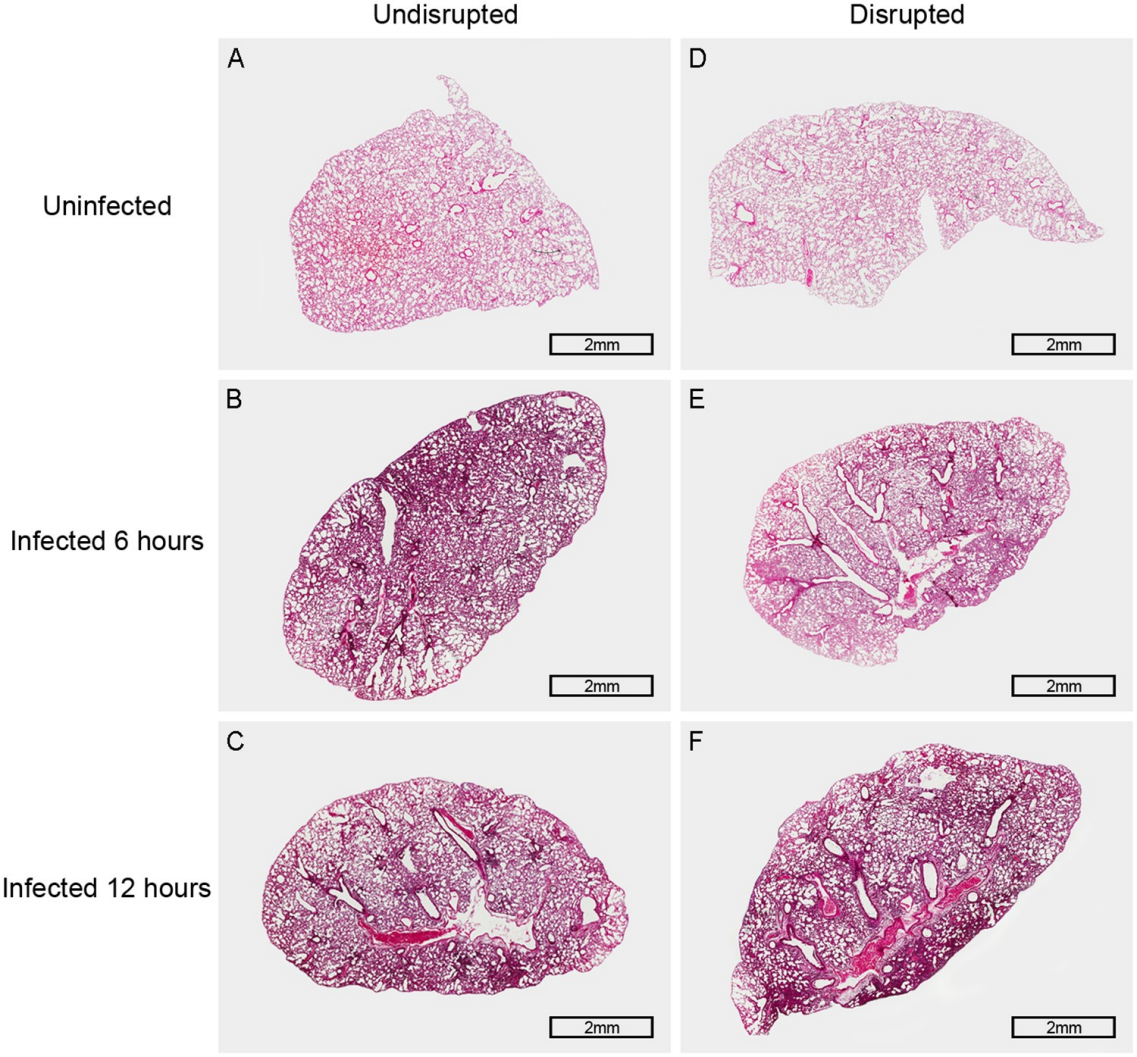
Histopathology of the lung in TLR4-deficient mice 6 hours and 12 hours after pneumococcal infection. Representative pictures of H & E stained lung tissues: (A) The picture of the lung tissue in gut microbiota-undisrupted uninfected mice (n = 6 per group). (B and C) Pictures of the lung tissue in gut microbiota-undisrupted mice 6 hours and 12 hours after infection (n = 6 per group). (D) The picture of the lung tissue in gut microbiota-disrupted uninfected mice (n = 6 per group). (E and F) Pictures of the lung tissue in gut microbiota-disrupted mice 6 hours and 12 hours after infection (n = 6 per group). Original magnification, × 20; scale bar = 2 mm.

To determine the effect of TLR4 on the host resistance against pneumococcal infection, wild-type and TLR4-deficient mice were infected intra-nasally with *S*. *pneumoniae*. The survival (Fig 3A: P < 0.001), pulmonary bacterial clearance (Fig 3B: P = 0.004, 6 hours; P = 0.011, 12 hours) and pulmonary cytokine levels (Fig 3C: P = 0.009, 6 hours. Fig 3D: P = 0.006, 6 hours; P = 0.013, 12 hours) were significantly reduced in the TLR4-deficient mice after *S*. *pneumoniae* infection compared to the wild-type mice. In addition, TLR4-deficient mice exhibited more serious lung histopathological injury (Fig 2A–2C and Fig 4A–4C). Taken together, loss of TLR4 reduced the host resistance to *S*. *pneumoniae*, indicating its involvement in the host immune response to pneumococcal infection.

### The pulmonary resistance to *S*. *pneumoniae* is decreased to a lesser extent in TLR4-deficient mice compared to the wild-type after gut microbiota disruption

**Fig 5:**
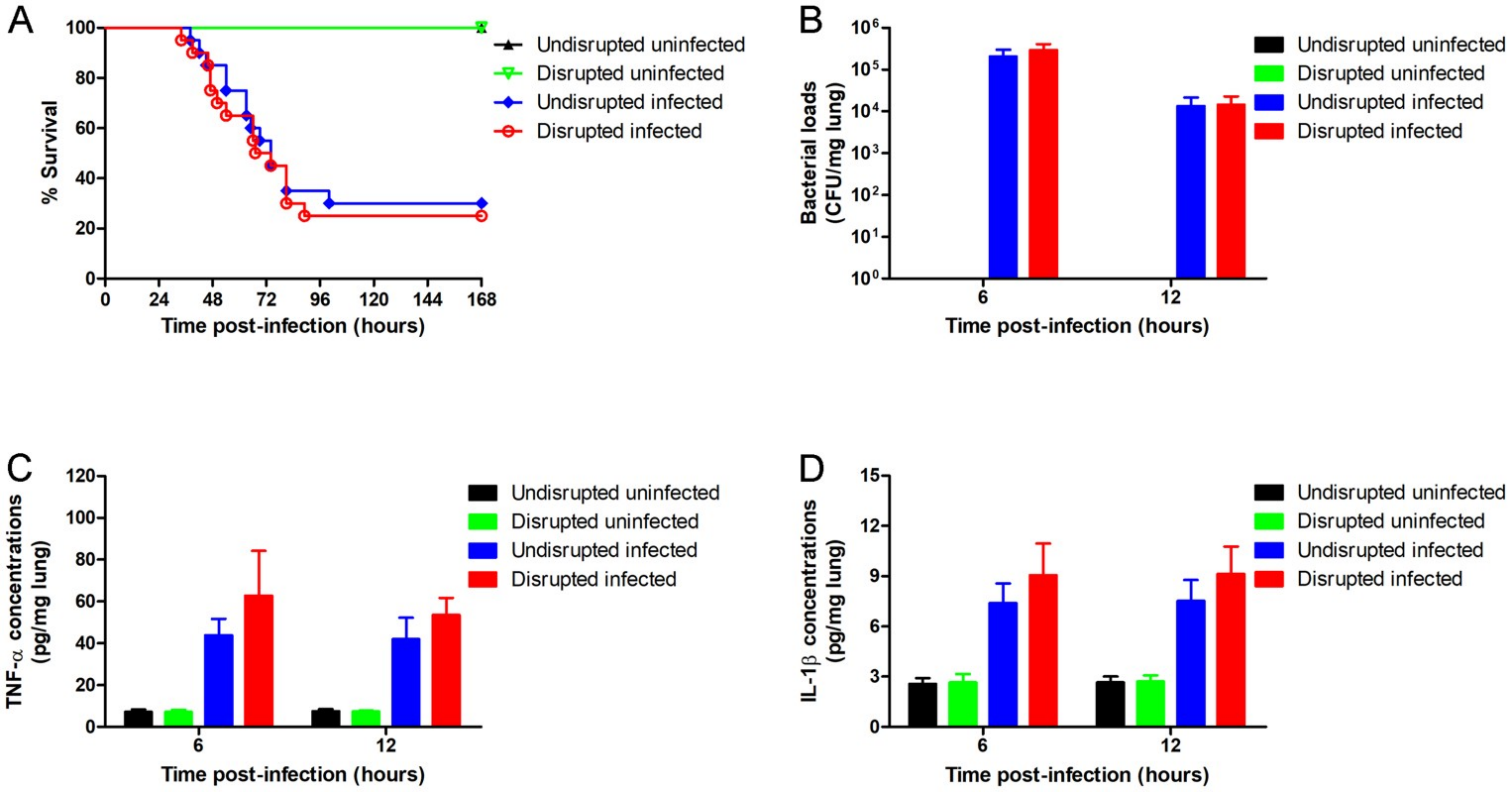
Gut microbiota disruption does not obviously affect the pulmonary resistance to *S*. *pneumoniae* in TLR4-deficient mice. TLR4-deficient mice were treated with broad-spectrum antibiotics or autoclaved saline, and infected intra-nasally with 2.3 × 10^8^ CFU of *S*. *pneumoniae*. (A) The survival was observed for 7 days (n = 20 per group). (B) Bacterial load, and (C) TNF-α and (D) IL-1β concentration per mg lung tissue were determined 6 hours and 12 hours after infection (n = 6 per group). CFU, colony-forming units. Data are presented as means ± SD. Analyzed with the log-rank test or unpaired Student’s t test.

To determine the role of TLR4 in the decreased resistance to *S*. *pneumoniae* infection following gut microbiota disruption, we first analyzed the effect of the latter on pulmonary resistance in TLR4-deficient mice. The gut microbiota-disrupted TLR4-deficient mice showed statistically similar survival (Fig 5A: P = 0.706), pulmonary bacterial clearance (Fig 5B: P = 0.167, 6 hours; P = 0.879, 12 hours), lung injury (Fig 4), and pulmonary levels of TNF-α and IL-1β (Fig 5C: P = 0.071, 6 hours; P = 0.055, 12 hours. Fig 5D: P = 0.095, 6 hours; P = 0.082, 12 hours) compared to the undisrupted controls, indicating that gut microbiota disruption did not obviously affect the pulmonary resistance to *S*. *pneumoniae* in TLR4-deficient mice.

Subsequently, we compared the degree of decline in pulmonary resistance to *S*. *pneumoniae* in wild-type and TLR4-deficient mice after gut microbiota disruption. While the survival of the wild-type mice (Fig 1A and Table 1) was decreased by 45% (P < 0.001) after gut microbiota disruption, that of TLR4-deficient mice (Fig 5A and Table 1) decreased only by 5% (P = 0.706). Similarly, the pulmonary bacterial load of gut microbiota-disrupted wild-type mice (Fig 1B and Table 2) increased by 3.48-fold after 6 hours (P = 0.001) and 6.95-fold after 12 hours (P = 0.016) of pneumococcal infection compared to the undisrupted controls, while that of TLR4-deficient mice (Fig 5B and Table 2) increased by only 0.42-fold (P = 0.167) and 0.05-fold (P = 0.879) at the respective time points. Histopathological examination of the lungs (Figs 2 and 4) showed more severe lung injury in the gut microbiota-disrupted wild-type mice compared to the undisrupted controls after pneumococcal infection, which was considerably less in the corresponding TLR4-deficient mice. Taken together, the degree of decline in pulmonary resistance was significantly lower in the TLR4-deficient mice compared to the wild-type mice after gut microbiota disruption.

**Table 1:** The effect of gut microbiota disruption on survival in wild-type and TLR4-deficient mice after pneumococcal infection.

	Undisrupted wild-type	Disrupted wild-type	Undisrupted TLR4-deficient	Disrupted TLR4-deficient
**Survival, % (n/n)**	100 (20/20)	55 (11/20)	30 (6/20)	25 (5/20)
**Change of survival, %**	45		5	

The survival of wild-type and TLR4-deficient mice after pneumococcal infection was determined according to Figs 1A and 5A respectively, and the change in survival rates between the gut microbiota-disrupted and undisrupted controls was calculated by the following formula:
Changeofsurvival=Survivalofundisruptedmice-Survivalofdisruptedmice

n/n, the number of mice alive/total number of mice observed; Change of survival, Change of survival between gut microbiota-disrupted mice and their undisrupted controls.

**Table 2:** The effect of gut microbiota disruption on pulmonary bacterial clearance in wild-type and TLR4-deficient mice after pneumococcal infection.

	Time post-infection (hours)	
	6	12
**Bacterial load of wild-type mice, CFU**		
Gut microbiota-undisrupted	2.70 × 10^4^ ± 1.99 × 10^4^	7.89 × 10^2^ ±4.10 × 10^2^
Gut microbiota-disrupted	1.21 × 10^5^ ±4.26 × 10^4^	6.27 × 10^3^ ±3.78 × 10^3^
Fold change of bacterial load	3.48	6.95
**Bacterial load of TLR4-deficient mice, CFU**		
Gut microbiota-undisrupted	2.07 × 10^5^ ±8.91 × 10^4^	1.36 × 10^4^ ±7.90 × 10^3^
Gut microbiota-disrupted	2.93 × 10^5^ ±1.10 × 10^5^	1.43 × 10^4^ ±8.45 × 10^3^
Fold change of bacterial load	0.42	0.05

Data are presented as means ± SD (n = 6 per group). Bacterial load of wild-type and TLR4-deficient mice after pneumococcal infection were determined according to Figs 1B and 5B respectively, and the fold change in bacterial load between gut microbiota-disrupted mice and their undisrupted controls was calculated by the following formula:
$$\text{Fold}\;\text{change}\;\text{of}\;\text{bacterial}\;\text{load}\;=\;\left({\text{Mean}}_{\text{disrupted}}-{\text{Mean}}_{\text{undisrupted}}\right)/{\text{Mean}}_{\text{undisrupted}}$$
Mean_disrupted_, Mean bacterial load of disrupted mice; Mean_undisrupted_, Mean bacterial load of undisrupted mice; Fold change of bacterial load, fold change of bacterial load between gut microbiota-disrupted mice and their undisrupted controls; CFU, colony-forming units.

## Discussion

Pulmonary infection of *S*. *pneumoniae* remains a major public health problem worldwide, and is associated with significant morbidity and mortality [43]. Recent studies show that the immunological effects of gut microbiota extend beyond the gastrointestinal tract [44]. Disruptions in the gut microbiota reduce pulmonary resistance against pathogens such as *S*. *pneumoniae* [13, 17]. Antibiotics are often used to study the role of gut microbiota in pulmonary defense [44]. In this study, we found that administering broad-spectrum antibiotics to mice disrupted their gut microbiota, both in terms of richness and species composition, which is consistent with previous studies [33]. Schuijt TJ *et al* showed that pulmonary resistance to *S*. *pneumoniae* was decreased in gut microbiota-disrupted mice compared to the undisrupted controls after pneumococcal infection [17]. In the present study, an *in vivo* experiment was made with TLR4-deficient mice to investigate whether TLR4 is involved in the reduced pulmonary resistance to *S*. *pneumoniae* caused by gut microbiota disruption. We compared the degree of decline in pulmonary resistance in the wild-type and TLR4-deficient mice after gut microbiota disruption, and found that it was lower in the latter in survival and pulmonary bacterial clearance. In addition, the severity of pulmonary injury had a similar tendency to above result—the gut microbiota-disrupted wild-type mice showed more severe lung injury, with thicker alveolar walls, inflammatory cellular infiltration and structural damage in the alveoli, compared to the undisrupted controls, while the gut microbiota-disrupted TLR4-deficient mice showed relatively minor structural damage in the alveoli. Collectively, these indicate that the deficiency of TLR4 reduces the difference of the resistance to pneumococcal infection between gut microbiota-disrupted mice and their undisrupted controls, and that the TLR4 pathway is likely associated with the decreased pulmonary resistance to *S*. *pneumoniae* caused by gut microbiota disruption.

The development of pulmonary innate immunity requires the recognition of the microbial ligands by the immune cells via surface-bound PRRs, and production of early response cytokines that further amplify host response to pathogens [45, 46]. These cytokines lead to local and systemic inflammatory responses and also activate the adaptive immune response [47]. The levels of TNF-α and IL-1β, two key pro-inflammatory cytokines, are therefore often tested following lung injury [47–52]. In the present study, the TLR4-deficient mice had lower TNF-α and IL-1β levels and decreased pulmonary resistance after *S*. *pneumoniae* infection compared to wild-type mice, indicating that the TLR4 pathway mediates the immune response against pneumococcal infection. This is consistent with previous studies that have explored the function of TLR4 in the immune response to *S*. *pneumoniae* [21, 25]. Mice infected with *S*. *pneumoniae*, *E*. *coli* and *K*. *pneumoniae* showed decreased TNF-α levels and elevated IL-1β levels after gut microbiota disruption [14, 15, 17]. In our study however, both TNF-α and IL-1β were upregulated in the lungs of gut microbiota-disrupted mice compared to the undisrupted controls after pneumococcal infection. Considering the decreased pulmonary bacterial clearance after gut microbiota disruption, it is clear that local cytokine release does not inhibit bacterial proliferation but instead stimulates their growth. In addition, the increased cytokine levels are the likely cause of the histopathological damage seen in the lungs after infection.

## Conclusions

The TLR4 pathway is involved in the reduced pulmonary resistance to *S*. *pneumoniae* caused by gut microbiota disruption, and therefore is a potential target for the management and control of pneumococcal infections.

## Supporting information

S1 Table Antibiotic administration had no effect on pneumococcal infection. Wild-type mice (n = 5) were given broad-spectrum antibiotics or autoclaved saline for 3 weeks. Three days after cessation of antibiotic administration, 0.2 ml sera, whole lungs or 0.3 g livers were homogenized in 3 ml sterile THY broth, centrifuged twice at 10000 × g for 10 minutes, and then filtered through a 0.22 μm cellulose acetate membrane. One hundred microliters of the bacterial culture (about 1.5 × 10^7^ CFU/ml) was diluted 10-fold with three kinds of homogenates respectively, and 0.2 ml of each of the diluted bacteria was added to each of the 96-well microplate and incubated at 37°C under 5% CO_2_ for 9 hours. The bacterial culture diluted 10-fold with THY broth was used as a positive control, and three kinds of homogenates and THY broth were used as negative controls. The optical density (OD) of each well was measured at 620 nm, and the data were analyzed with the Student’s t test.
(XLSX)

S2 Table The altered diversity of gut microbiota in wild-type mice after antibiotic treatment. Wild-type mice were given broad-spectrum antibiotics (ampicillin, 10 mg/ml; neomycin sulfate, 10 mg/ml; metronidazole, 5 mg/ml) or autoclaved saline for 3 weeks by oral gavage. Fresh feces were collected, and the bacterial 16S rRNA genes were sequenced. The richness estimators (ACE and Chao) and diversity indices (Shannon and Simpson) were calculated. Antibiotics 1, feces collected 3 weeks after antibiotic treatment (n = 5 per group); Antibiotics 2, feces collected 3 days after cessation of antibiotic treatment (n = 5 per group); Saline, feces collected 3 days after cessation of saline (n = 5 per group); Coverage, the Good’s coverage; Sobs, the observed richness; ACE, abundance-based coverage estimator; Chao, bias-corrected Chao 1 richness estimator; Shannon, the Shannon-Weaver diversity index; Simpson, the Simpson diversity index. Data are presented as means ± SD. **P < 0.01, ***P < 0.001, analyzed with the Student’s t test.
(XLSX)

S3 Table The altered diversity of gut microbiota in TLR4-deficient mice after antibiotic treatment. TLR4-deficient mice were given broad-spectrum antibiotics (ampicillin, 10 mg/ml; neomycin sulfate, 10 mg/ml; metronidazole, 5 mg/ml) or autoclaved saline for 3 weeks by oral gavage. Fresh feces were collected, and the bacterial 16S rRNA genes were sequenced. The richness estimators (ACE and Chao) and diversity indices (Shannon and Simpson) were calculated. Antibiotics 1, feces collected 3 weeks after antibiotic treatment (n = 5 per group); Antibiotics 2, feces collected 3 days after cessation of antibiotic treatment (n = 5 per group); Saline, feces collected 3 days after cessation of saline (n = 5 per group); Coverage, the Good’s coverage; Sobs, the observed richness; ACE, abundance-based coverage estimator; Chao, bias-corrected Chao 1 richness estimator; Shannon, the Shannon-Weaver diversity index; Simpson, the Simpson diversity index. Data are presented as means ± SD. **P < 0.01, ***P < 0.001, analyzed with the Student’s t test.
(XLSX)

S4 Table Gut microbiota composition of the antibiotic-treated and untreated wild-type mice at the phylum level. The data are the proportion of the different phyla in the gut microbiota. Antibiotics 1, feces collected 3 weeks after antibiotic treatment; Antibiotics 2, feces collected 3 days after cessation of antibiotic treatment; Saline, feces collected 3 days after cessation of saline.
(XLSX)

S5 Table Gut microbiota composition of the antibiotic-treated and untreated TLR4-deficient mice at the phylum level. The data are the proportion of the different phyla in the gut microbiota. Antibiotics 1, feces collected 3 weeks after antibiotic treatment; Antibiotics 2, feces collected 3 days after cessation of antibiotic treatment; Saline, feces collected 3 days after cessation of saline.
(XLSX)

S6 Table Survival of mice after *S*. *pneumoniae* infection. All animals died before meeting the criteria for euthanasia. Time, Survival time; 0, Censored subject; 1, Death/Event.
(XLSX)

S7 Table Bacterial load in the lungs of mice after *S*. *pneumoniae* infection. CFU, colony-forming units.
(XLSX)

S8 Table TNF-α levels in the lungs of mice after *S*. *pneumoniae* infection. (XLSX)

S9 Table IL-1β levels in the lungs of mice after *S*. *pneumoniae* infection. (XLSX)

S1 Fig Results of statistical tests for gut microbiota composition between the antibiotic-treated and untreated wild-type mice at the phylum level. Wild-type mice were given broad-spectrum antibiotics (ampicillin, 10 mg/ml; neomycin sulfate, 10 mg/ml; metronidazole, 5 mg/ml) or autoclaved saline. The 16S rRNA genes were sequenced, and the composition of gut microbiota was compared at the phylum level (A and B). Antibiotics 1 (S1A Fig, blue), feces collected 3 weeks after antibiotic treatment (n = 5 per group); Antibiotics 2 (S1B Fig, blue), feces collected 3 days after cessation of antibiotic treatment (n = 5 per group); Saline (S1 Fig, red), feces collected 3 days after cessation of saline (n = 5 per group). Data are presented as means ± SD. *P < 0.05, **P < 0.01, ***P < 0.001, analyzed with Student’s t test.
(TIF)

S2 Fig Results of statistical tests for gut microbiota composition between the antibiotic-treated and untreated TLR4-deficient mice at the phylum level. TLR4-deficient mice were given broad-spectrum antibiotics (ampicillin, 10 mg/ml; neomycin sulfate, 10 mg/ml; metronidazole, 5 mg/ml) or autoclaved saline. The 16S rRNA genes were sequenced, and the composition of gut microbiota was compared at the phylum level (A and B). Antibiotics 1 (S2A Fig, blue), feces collected 3 weeks after antibiotic treatment (n = 5 per group); Antibiotics 2 (S2B Fig, blue), feces collected 3 days after cessation of antibiotic treatment (n = 5 per group); Saline (S2 Fig, red), feces collected 3 days after cessation of saline (n = 5 per group). Data are presented as means ± SD. *P < 0.05, ***P < 0.001, analyzed with Student’s t test.
(TIF)
