# Supplementary figures and images for: TLR4 deficiency reduces pulmonary resistance to Streptococcus pneumoniae in gut microbiota-disrupted mice
Source: PLoS One. 2018 Dec 18;13(12):e0209183. doi: 10.1371/journal.pone.0209183 (PMC6298678; doi:10.1371/journal.pone.0209183)

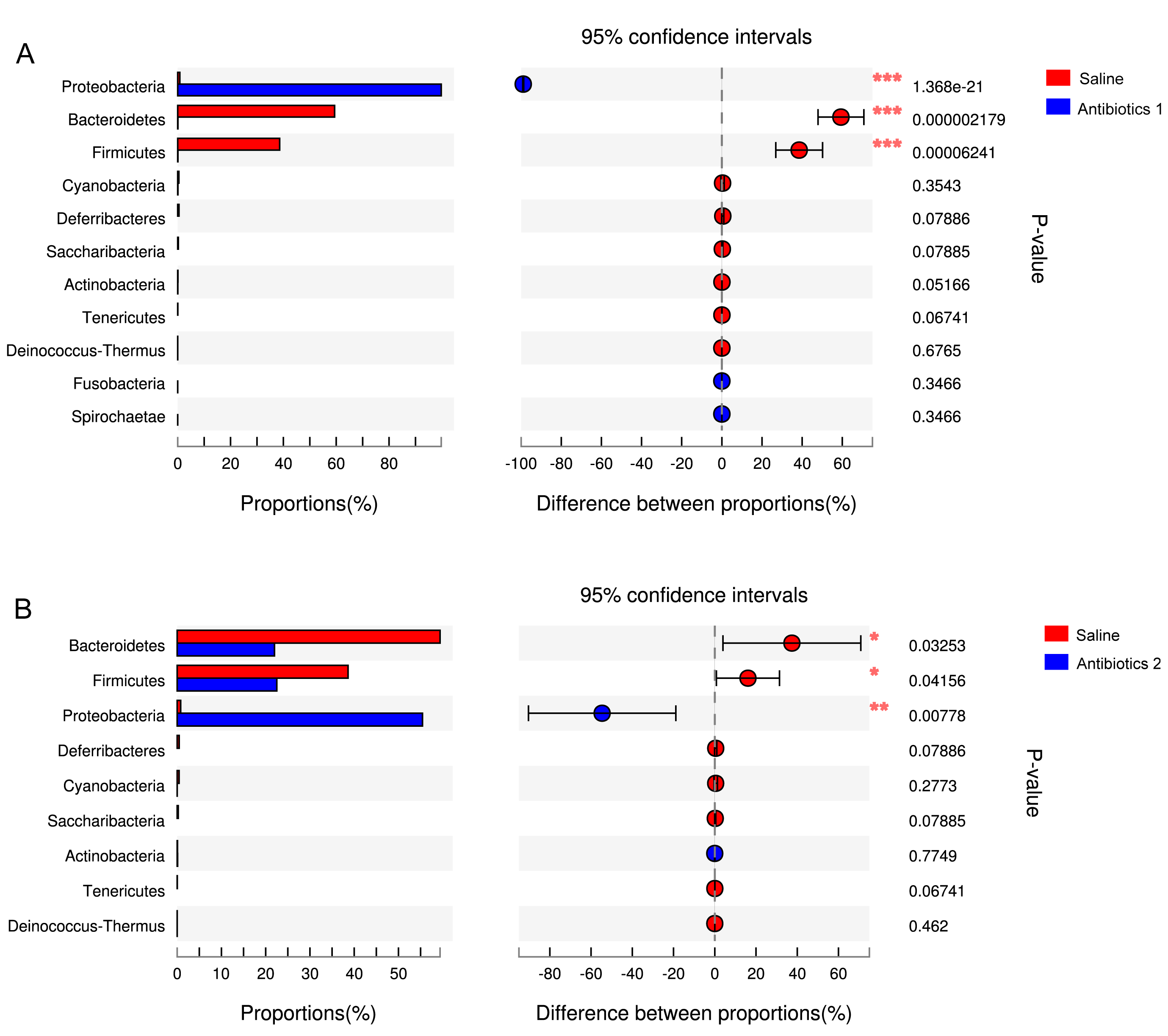

Supplement: S1 Fig — Wild-type mice were given broad-spectrum antibiotics (ampicillin, 10 mg/ml; neomycin sulfate, 10 mg/ml; metronidazole, 5 mg/ml) or autoclaved saline. The 16S rRNA genes were sequenced, and the composition of gut microbiota was compared at the phylum level (A and B). Antibiotics 1 (S1A Fig, blue), feces collected 3 weeks after antibiotic treatment (n = 5 per group); Antibiotics 2 (S1B Fig, blue), feces collected 3 days after cessation of antibiotic treatment (n = 5 per group); Saline (S1 Fig, red), feces collected 3 days after cessation of saline (n = 5 per group). Data are presented as means ± SD. *P < 0.05, **P < 0.01, ***P < 0.001, analyzed with Student’s t test. (TIF) [file pone.0209183.s010.tif]

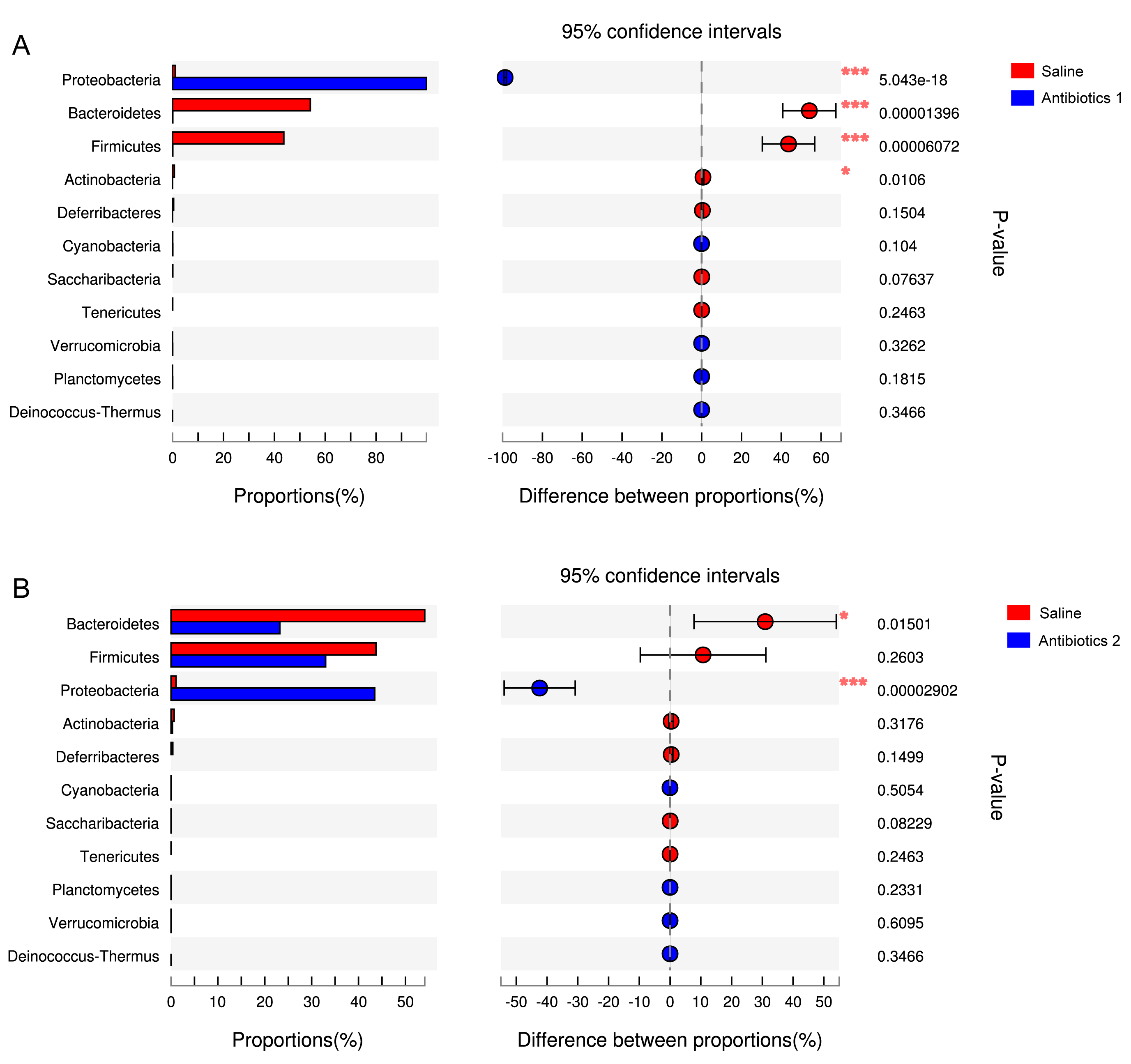

Supplement: S2 Fig — TLR4-deficient mice were given broad-spectrum antibiotics (ampicillin, 10 mg/ml; neomycin sulfate, 10 mg/ml; metronidazole, 5 mg/ml) or autoclaved saline. The 16S rRNA genes were sequenced, and the composition of gut microbiota was compared at the phylum level (A and B). Antibiotics 1 (S2A Fig, blue), feces collected 3 weeks after antibiotic treatment (n = 5 per group); Antibiotics 2 (S2B Fig, blue), feces collected 3 days after cessation of antibiotic treatment (n = 5 per group); Saline (S2 Fig, red), feces collected 3 days after cessation of saline (n = 5 per group). Data are presented as means ± SD. *P < 0.05, ***P < 0.001, analyzed with Student’s t test. (TIF) [file pone.0209183.s011.tif]
